# Efficient Hot Carrier Management in Lead Halide Perovskite Micro‐Disks Revealed by Super‐Diffusive Migration

**DOI:** 10.1002/advs.76474

**Published:** 2026-07-13

**Authors:** Nithin Pathoor, Shun Omagari, Martin Vacha

**Affiliations:** ^1^ Department of Materials Science and Engineering Institute of Science Tokyo Meguro‐ku Tokyo Japan

**Keywords:** carrier super‐diffusion, hot‐carrier stabilization, large polarons, lead halide perovskites

## Abstract

Metal halide perovskites exhibit exceptional optoelectronic properties arising from their defect tolerance, high absorption coefficient, tunable bandgap, and long‐range charge transport. The soft crystal structure and interaction with light lead to a number of interesting observations over the years. Here, we investigate carrier migration in anti‐solvent‐assisted grown methylammonium lead bromide (MAPbBr_3_) micro‐disks (MDs) using spatially resolved photoluminescence (PL) lifetime microscopy. Confocal excitation reveals rapid outward carrier migration accompanied by pronounced non‐linear PL spatial broadening, indicative of super‐diffusive carrier migration. Under deep conduction band excitation at 375 nm, carriers exhibit accelerated diffusion over hundreds of nanometers on an ultra‐stable nanosecond timescale, with diffusivity increasing from initial average values of ∼0.44 cm^2^/s to equilibrium values exceeding 0.88 cm^2^/s. The degree of non‐linearity, represented by an average non‐linearity coefficient of 1.5, signifies strong deviation from classical diffusion. Comparative measurements with 485 nm excitation, temperature‐dependent studies, and analysis of diffusivity gain and non‐linearity demonstrate that these dynamics originate from stabilized hot carriers interacting with low‐energy LO phonon modes through large‐polaron formation. The observation of nanosecond‐scale super‐diffusion in perovskite microstructures highlights efficient hot‐carrier transport, with implications for designing high‐performance optoelectronic and hot‐carrier energy‐harvesting devices.

## Graphical abstract

Hot‐carrier transport in metal halide perovskites is investigated using spatiotemporally‐resolved photoluminescence microscopy. Mesa‐shaped MAPbBr_3_ microdisks exhibit anomalous super‐diffusive carrier propagation on nanosecond timescales, driven by hot‐carrier stabilization and strong carrier–phonon coupling. The results reveal unconventional carrier transport beyond classical diffusion, with implications for hot‐carrier optoelectronic and energy‐conversion applications.

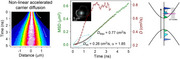

## 1 Introduction

Metal halide perovskites have emerged as a versatile class of semiconductors with broad applications in photovoltaics, light‐emitting diodes, photodetectors, and nonlinear optics [1, 2, 3, 4, 5, 6]. Even thin‐film perovskite devices composed of meso‐length‐scale crystal grains enable low energy losses and efficient extraction of charges generated by absorption across the visible to infrared regions of the electromagnetic spectrum. The facile synthesis, high absorption coefficients and photoluminescence (PL) quantum yields, compositionally tunable bandgaps, slow electron–hole recombination, long carrier diffusion lengths, and defect‐tolerant electronic structure distinguish perovskites from conventional semiconductors [7, 8, 9]. In addition, the soft ionic lattice and mixed organic–inorganic composition provides unique pathways for charge–lattice interactions not present in classical covalent materials [10, 11, 12]. The structural flexibility and ferroelectric domains associated with organic cations have been shown to facilitate charge separation through internal junction formation [13]. However, the soft lattice also introduces stability challenges, including phase separation, photo‐induced and moisture‐mediated defect formation and degradation [14, 15, 16, 17]. On the other hand, perovskites have been reported to exhibit self‐healing behavior in which material properties can partially recover, a phenomenon linked to ion migration and lattice softness [18].

A major objective in perovskite optoelectronics research is minimizing energy losses to convert the majority of the absorbed photons into usable electrical energy. Numerous strategies are being actively explored to enhance both the stability and performance of lead halide perovskites [19, 20]. These include tandem architectures employing perovskite‐perovskite or perovskite silicon junctions, utilization of multiple carrier generation processes, and suppression of hot‐carrier thermalization losses to improve light harvesting efficiency [21, 22, 23]. At the material level, grain boundaries and defects within photoactive layers represent major sources of structural heterogeneity and nonradiative recombination, directly impacting carrier transport and extraction. Grain‐boundary treatments using ligands or antisolvents have been shown to improve device performance by modifying film morphology, enhancing hydrophobicity, and passivating defects, thereby promoting uniform crystal growth and improved film quality [24, 25, 26, 27, 28]. Consequently, understanding both structural and functional heterogeneity is essential for optimizing perovskite materials and device performance [29]. In this context, fluorescence microscopy offers a powerful approach, providing combined spatial, temporal, and spectral resolution to probe heterogeneity, identify nonradiative recombination pathways, and assess carrier transport behavior across multiple length scales [29, 30].

Efficient carrier transport is a key attribute underpinning the success of perovskite‐based optoelectronic devices. The efficiency of the transport is enabled by long carrier lifetimes and diffusion lengths that often exceed the dimensions of typical photoactive layers [31, 32]. Long‐range carrier diffusion is important for effective charge collection at external contacts [15, 33]. Correlated PL blinking across multiple grains demonstrates efficient carrier migration despite the presence of defects and grain boundaries in thin films [29]. Secondary processes such as photon recycling and light trapping via waveguiding further enhance the apparent carrier diffusion and contribute to improved device performance [34, 35]. A detailed understanding of excited carrier dynamics is therefore critical for correlating material properties with device functionality. These processes include understanding carrier excitation, thermalization and cooling toward the conduction band minimum, radiative or non‐radiative recombination, carrier transport and charge extraction [36, 37]. In this work, we investigate carrier diffusion dynamics in mesa‐shaped methylammonium lead bromide (MAPbBr_3_) micro‐disks (MDs) using spatially resolved time‐correlated single‐photon counting measurements. We report and analyze an anomalous accelerated carrier propagation observed in these microstructures.

## 2 Results

**FIGURE 1:**
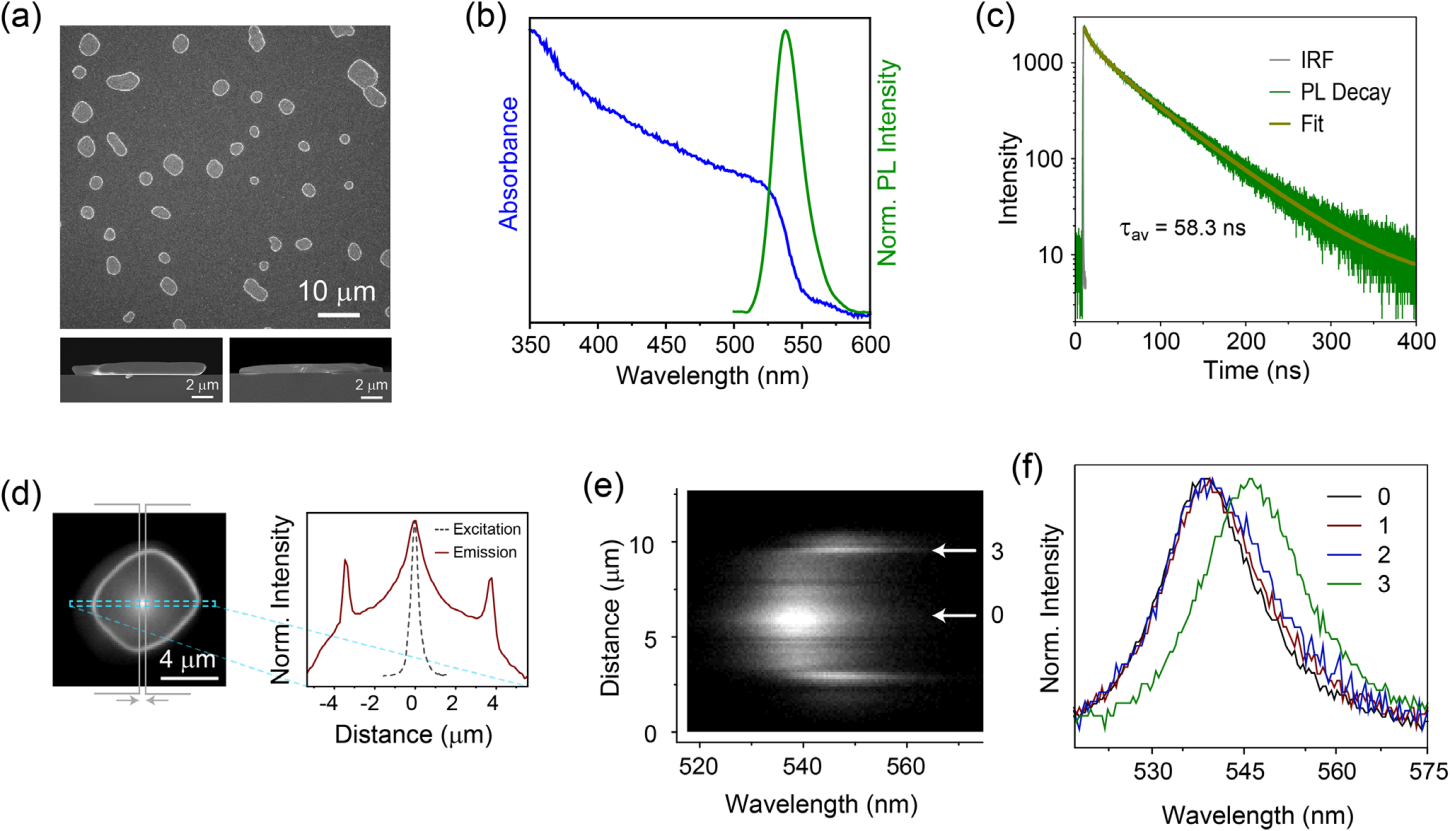
Morphological and optical characterization of MAPbBr_3_ microdisks (MDs). (a) SEM images of MDs; the bottom panel displays cross‐sectional SEM images of two representative MDs. (b) Bulk, solid‐state absorption and PL spectra of thin film of MDs. (c) Time‐resolved PL decay curve of the thin film, fitted with a tri‐exponential function with lifetime components: τ_1_ = 65 ns, τ_2_ = 22 ns, and τ_3_ = 4.4 ns. (d) PL image of an MD under confocal excitation, and its emission intensity cross‐section along with excitation profile. (e) Spatially resolved PL spectral mapping with (f) representative spectra recorded at the excitation site and distal locations.

The MAPbBr_3_ MDs grown on microscope coverslips exhibit quasi‐circular or deformed rectangular shapes (Figure 1a), with lateral dimensions ranging from one to several micrometers. Cross‐sectional SEM imaging shows that their thickness is on the order of few hundred nanometers. Absorption and PL spectra are typical of bulk MAPbBr_3_ (Figure 1b), including a substantial absorption‐emission overlap. The time‐correlated single‐photon counting measurements reveal a PL decay with an average lifetime of 58.3 ns. Efficient long‐range carrier migration is evident from the spatially broad emission observed across the entire MD under localized confocal excitation (Figure 1d). The PL intensity decreases with distance from the excitation spot but becomes enhanced again near the disk edges. This behavior is consistent with the mesa‐shaped MD acting as a parallel plane waveguide, wherein a portion of the emitted photons is guided laterally and subsequently scatters out at the MD boundaries [38]. Because of the strong spectral overlap between absorption and emission, some of the guided photons, particularly those at higher energies, are reabsorbed, resulting in a red‐shifted emission at the edges (Figure 1e,f). In contrast, no significant spectral shift is detected within the interior region of the MD, indicating effective transport with only a minor contribution from the reabsorption and re‐emission processes.

**FIGURE 2:**
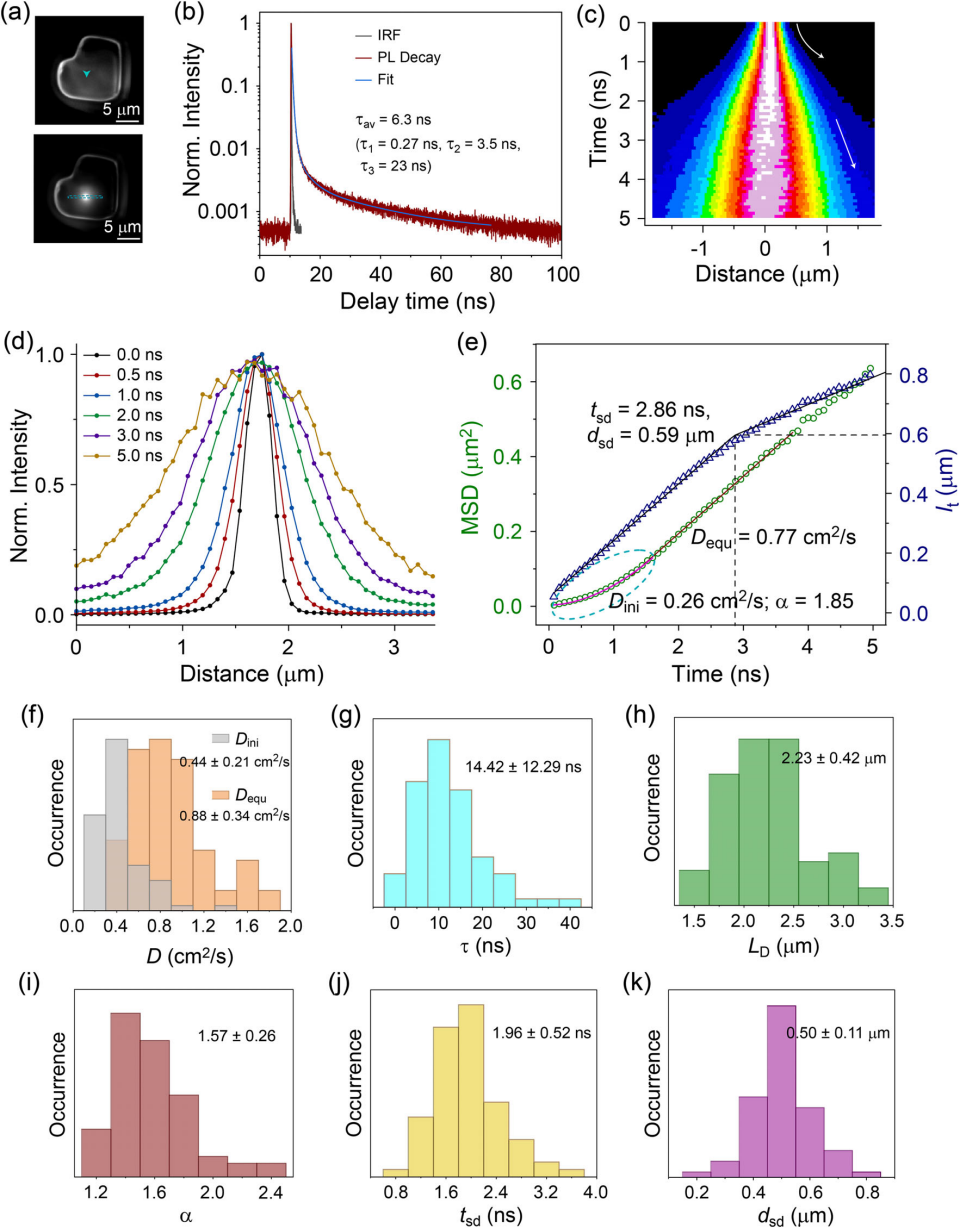
Quantitative carrier diffusion analysis of MAPbBr_3_ MDs. (a) PL images of a MAPbBr_3_ MD under widefield (top) and confocal (bottom) excitation. (b) The PL decay measured at the excitation location of the MD. (c) The spatiotemporal evolution of PL intensity profile across the excitation location, and (d) representative cross‐sectional profiles at the pulse arrival time (*t*_0_) and subsequent delay times. (e) Mean square displacement (MSD) plot and the corresponding transport length plot for the MD. The intersection of the two linear fitting regions in the transport length plot quantifies the super‐diffusive transport time (*t*_sd_) and distance (*d*_sd_). Statistical distributions (N = 75 MDs) of (f) initial and equilibrium diffusion coefficients, (g) average PL lifetimes (h) carrier diffusion lengths, (i) non‐linearity coefficient, α, (j) super‐diffusive transportation time, and (k) transportation distance.

The excited carrier diffusion in the MDs was characterized using spatially resolved PL lifetime measurement. A 375 nm pulsed laser was used to generate carriers deep within the conduction band. A wide‐field PL image of a representative MD is shown in Figure 2a, top, and the same MD under confocal excitation in Figure 2a, bottom. The strong intensity at the edges arises partly from the waveguided photons. To accurately probe carrier diffusion, only the interior region of the MD was analyzed. A high magnification (∼ 430x) was employed to meet the Nyquist sampling criterion for the spatially resolved PL lifetime measurements using an avalanche photodiode (APD). When the APD is centered for detection of the emission from the excitation site (∼ 115 nm × 115 nm area in the sample), the PL decay exhibits a multi‐exponential profile with an average lifetime of 6.3 ns, indicative of the rapid carrier migration away from the excitation volume. Carriers that recombine radiatively at locations farther from the excitation show longer lifetimes (Figure S1). This allows us to measure the carrier diffusivity through spatial lifetime variations. By scanning the APD across the confocally excited region of the MD, a two‐dimensional map of the temporal evolution of PL intensity (Figure 2c) was obtained. At early times, the emission profile closely matches the excitation distribution and reflects predominantly local radiative recombination. At longer delays, the PL profile broadens due to carrier diffusion. Interestingly, this broadening is not consistent with free diffusion; instead, an accelerated carrier population expansion is observed, suggesting non‐linear diffusion dynamics with an additional carrier transport mechanism within the MDs.

To quantify this behavior, we extracted PL cross‐section profiles at specific times; several representative profiles are shown in Figure 2d. Each PL cross‐section was fitted with a Gaussian function, and the mean square displacement (MSD) was calculated from the variance of these fits [39]. The resulting MSD as a function of delay time (Figure 2e, green scatter plot) shows a distinctly non‐linear trend, deviating from the linear dependence expected for free diffusion. The upward truncation indicates a super‐diffusive transport regime, in which carriers undergo accelerated migration during the initial few nanoseconds (Figure S1c). Fitting the early‐time MSD data with a diffusion model yields a super‐diffusive trend with an initial diffusion coefficient *D*_ini_ = 0.29 cm^2^/s and a truncation coefficient α = 1.7, significantly exceeding the value of 1 associated with normal diffusion. At longer delay times, carriers transition to a linear behavior corresponding to an equilibrium diffusivity *D*_equ_ = 0.77 cm^2^/s. The degree of carrier acceleration or super‐diffusion is expressed by both the α value (larger than 1) and by increased *D*. The evolution of carrier transport length *l*_t_ is quantified by the relative broadening of carrier distribution (defined in the Materials and methods section, Supporting Information) (Figure 2e, blue scatter plot) shows dependence within the super‐diffusive regime. Overall, the accelerated carrier propagation for the MD occurs over a timescale of a few nanoseconds and spans distances of a few hundred nanometers.

The results are reproduced on multiple MDs, with two additional representative datasets provided in Figure S2. The well‐separated distributions of initial and equilibrium diffusivities, with the average equilibrium value (*D*_equ_ = 0.88 cm^2^/s) nearly twice the initial diffusivity (*D*_ini_ = 0.44 cm^2^/s), provide further evidence of pronounced super‐diffusive carrier transport in these crystals. A statistical average PL lifetime τ_av_ of 14.2 ns was recorded at the excitation locations (Figure 2g) and used to estimate the carrier diffusion lengths *L*_D_ as $L_{D}=\sqrt{\tau_{\text{av}}D_{\text{equ}}}$. The resulting distribution (Figure S3) shows an average diffusion length of 1 µm; however, this value is likely underestimated because the lifetime at the excitation spot shortened due to carrier escape is used for the calculation. To obtain a more accurate estimate of the carrier diffusion length, the average bulk lifetime (τ_bulk_ = 58.3 ns) was used in the calculation. The resulting diffusion‐length distribution (Figure 2h) exhibits an average value of 2.23 µm, more than twice the initially estimated value of ∼1 µm. Another characteristic parameter, α (Figure 2i), shows more than a 50% increase relative to the free‐diffusion value of 1, with an average α = 1.57. Such super‐diffusive carrier diffusion is typically associated with ballistic transport of hot carriers [39, 40]. While this type of non‐equilibrium transport can facilitate long‐range carrier motion within photo‐active layers, it has been reported to occur on femtosecond timescales, driven by phonon scattering. The timescale of super‐diffusive transport (*t*_sd_) is defined as the time at which the linear increase of the carrier transport length (*l*_t_) transitions to a saturated regime. To determine *t*_sd_, the *l*_t_ trajectory was fitted with two linear functions corresponding to the super‐diffusive and saturation regimes, and the intersection of these fits was taken as *t*_sd_. The corresponding super‐diffusive transport length (*d*_sd_) was defined as the carrier transport distance at *t*_sd_. The super‐diffusive transportation time *t*_sd_ extracted here (Figure 2j; defined in Figure 2e) yields a much longer average value of 1.96 ns, resulting in an average super‐diffusive transportation distance *d*_sd_ of 500 nm (Figure 2k; defined in Figure 2e). Although metal halide perovskites are known to sustain hot carriers significantly longer than conventional semiconductors, the observation of super‐diffusion on the nano‐second timescale is surprising.

**FIGURE 3:**
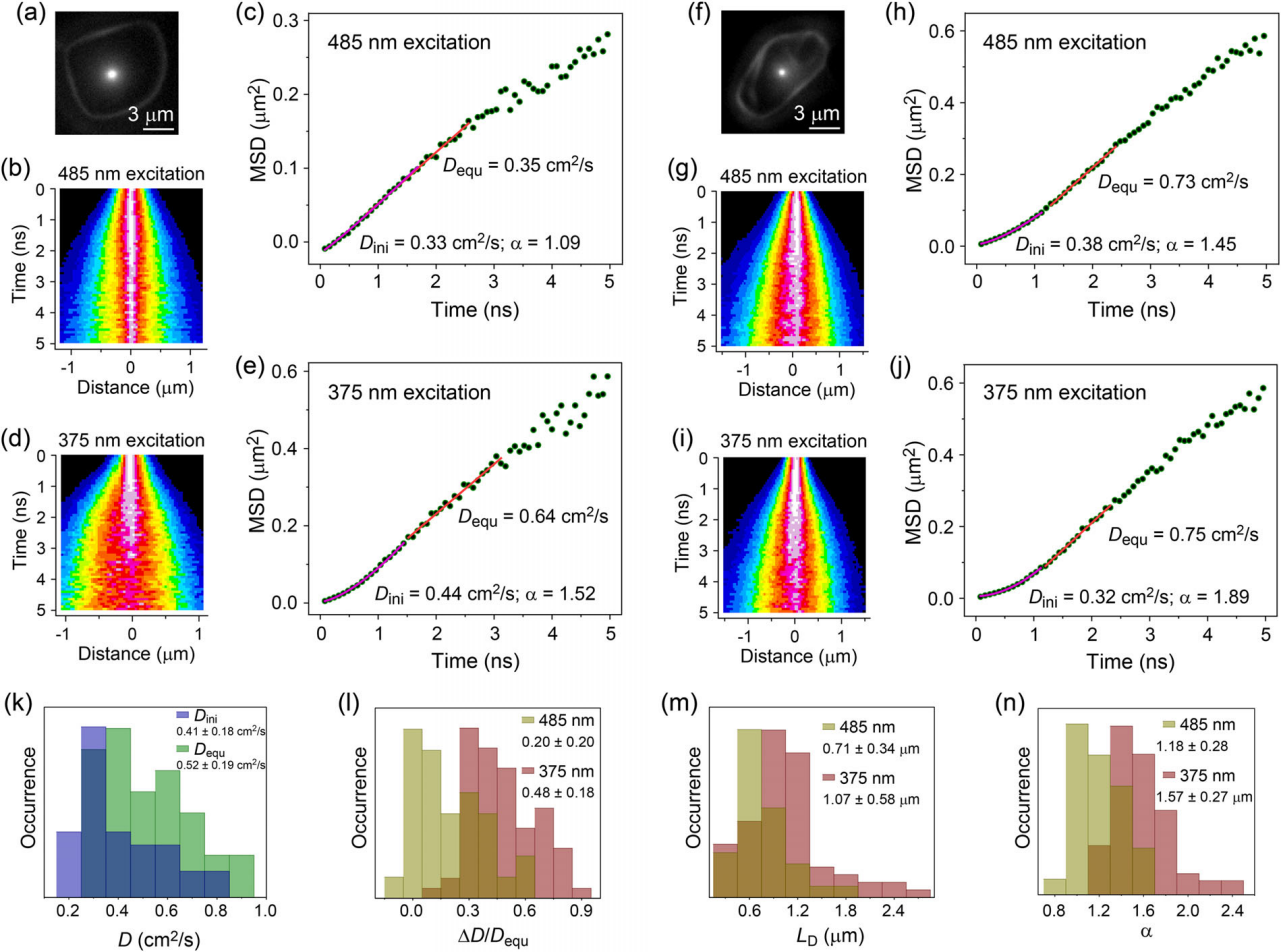
Wavelength dependence of carrier super‐diffusivity. (a) PL image and (b) spatiotemporal PL intensity evolution under 485 nm pulsed confocal excitation. (c) Corresponding MSD plot showing a nearly linear trend over the delay time. (d) PL cross‐section evolution and (e) MSD plot of the same MD under 375 nm excitation, demonstrating carrier super‐diffusion. (f) PL image, (g) PL cross‐section evolution, and (h) MSD plot for another MD with 485 nm excitation, showing super‐diffusion. (i) PL cross‐section profile and (j) MSD plot for the same MD under 375 nm excitation showing enhanced super‐diffusivity. (k) The statistical distributions of *D*_ini_ and *D*_equ_ for MDs under 485 nm excitation (N = 50). Comparison of the distributions for 485 nm and 375 nm excitations regarding (l) diffusivity gain, (m) carrier diffusion length, and (n) non‐linearity coefficients.

To confirm the involvement of hot carriers, we performed additional measurements under 485 nm (2.56 eV) excitation, which still generates hot carriers but not as deep in the conduction band as the 375 nm (3.31 eV) excitation. Under the 485 nm excitation, the PL cross‐section profile of a MD (PL image shown in Figure 3a) exhibits near‐linear spatial broadening over time (Figure 3b). This is reflected in the MSD evolution, where the extracted α value (1.09) is close to unity, and the equilibrium diffusivity (*D*_equ_ = 0.35 cm^2^/s) shows only a nominal increase relative to the initial *D*_ini_ (0.33 cm^2^/s). In contrast, excitation with 375 nm light on the same MD produces significantly deeper hot carriers, resulting in markedly non‐linear PL profile broadening and truncated MSD evolution (Figure 3d,e). Nevertheless, MDs can still display super‐diffusive behavior under 485 nm excitation in certain cases. An example is shown in Figure 3f–h, where *D*_ini_ = 0.38 cm^2^/s increases to *D*_equ_ = 0.73 cm^2^/s, accompanied by an elevated α = 1.45. When the same MD is excited with 375 nm laser, the degree of super‐diffusion becomes more pronounced, yielding α = 1.89 and diffusivities *D*_ini_ = 0.32 cm^2^/s and *D*_equ_ = 0.75 cm^2^/s. The α parameter exhibits a broad distribution of values and, particularly under 485 nm excitation, can approach unity, corresponding to free‐diffusive transport. The lateral dimensions of the MDs span a wide range, from a few to several micrometers, as shown in Figure S4, with crystal thicknesses ranging from approximately 500 nm to 1 µm. However, no clear correlation is observed between the lateral dimensions of the MDs and the degree of super‐diffusion (Figure S5), indicating that factors other than crystal size may contribute to the observed variations in transport behavior. Cross‐sectional SEM images (Figure S4) reveal pronounced grain boundaries in some crystals and even within individual MDs. These local structural inhomogeneities can introduce regions of elevated defect density, which may impede hot‐carrier transport and reduce the extent of super‐diffusion. Such defect‐mediated variations in carrier transport likely contribute to the observed heterogeneity among MDs, particularly under 485 nm excitation, where the super‐diffusive behavior is generally weaker.

The distributions of *D*_ini_ and *D*_equ_ (Figure 3k) show greater overlap under 485 nm excitation, with average values of 0.41 and 0.52 cm^2^/s, respectively. To quantify super‐diffusion, we define the diffusivity gain $\frac{\Delta D}{D_{\mathrm{equ}}}=\frac{\left(D_{\mathrm{equ}}-D_{\mathrm{ini}}\right)}{D_{\mathrm{equ}}}$. The value of diffusivity gain is substantial for 375 nm excitation (0.48) compared to 485 nm excitation (0.20) (Figure 3l). This difference also influences the carrier diffusion lengths, which vary with the excitation energy (Figure 3m). The enhanced super‐diffusivity is further evident in the distributions of α values for the two excitation sources (Figure 3n). While 375 nm laser produces a shallower excitation profile than 485 nm, the absorption depth remains sufficiently large to generate a substantial carrier population within the bulk of the MDs. The enhanced super‐diffusion under 375 nm excitation is therefore attributed primarily to higher hot‐carrier energies rather than surface‐confined transport. The strong dependence on excitation energy clearly implicates hot carriers’ involvement in the origin of the observed non‐linear carrier diffusion.

## 3 Discussion

**FIGURE 4:**
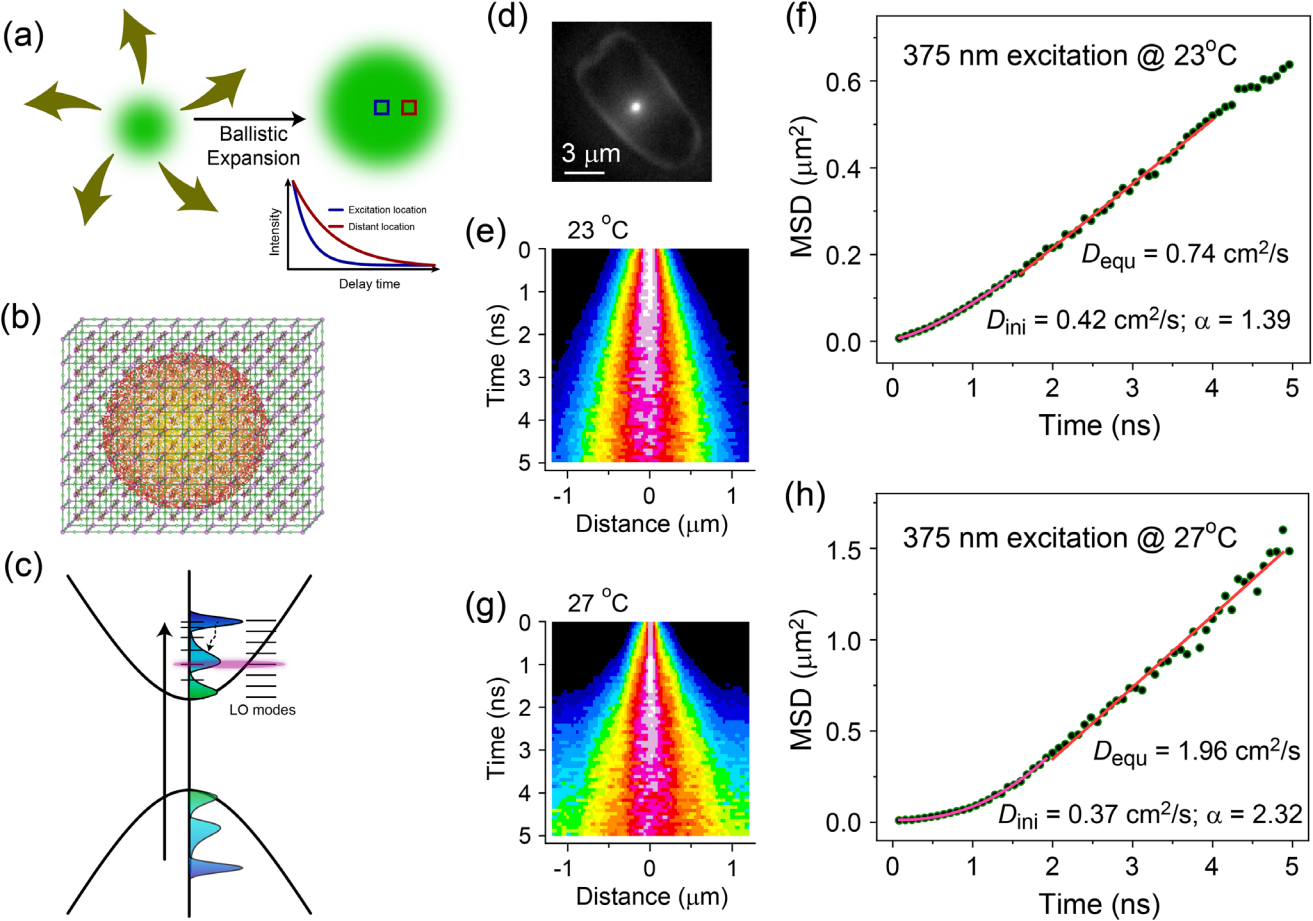
(a) Schematics illustrating accelerated carrier diffusion and the corresponding enhancement of PL lifetimes at distal locations. (b) Representation of large polaron formation upon photoexcitation. (c) Schematic diagram of carrier dynamics and hot‐carrier stabilization mediated by resonance with longitudinal optical (LO) phonon modes. (d) PL image and (e) spatiotemporal PL intensity evolution of a MD under 375 nm confocal excitation at room temperature (23°C), with the corresponding mean squared displacement (MSD) plotted in (f). Elevated temperature (27°C) results in enhanced super‐diffusion, evidenced by (h) PL intensity evolution and (i) MSD curve as a function of delay time.

Confocal excitation of an MD generates a population of excited carriers that rapidly migrate outward from the excitation spot (Figure 4a). The slow cooling of hot carriers sustains this outward transport on the nanosecond timescale. As a result, the PL intensity at the sub‐diffraction excitation region decays more rapidly (PL decay in Figure 2b, collected from 115 × 115 nm region) compared to the bulk PL decay (Figure 1c). In contrast, the PL lifetime recorded at positions distant from the excitation site increases due to the rapid hot carrier migration. Because the carrier diffusion length depends on both diffusivity and lifetime, the shortened lifetime measured at the excitation location, caused by the fast carrier escape, leads to an underestimated diffusion length. The accelerated carrier diffusion observed under deep band excitation (375 nm) indicates stabilization of hot carriers on nanosecond timescales. Sustaining long‐lived hot carriers is one of the core requirements for hot‐carrier solar cells, which promise increased power conversion efficiencies [41]. By harvesting hot‐carriers before cooling, such device can achieve higher photovoltages, thus utilizing energy that would otherwise be lost by heat dissipation, and provide a pathway to surpass the Shockley−Queisser limit [42, 43, 44]. In conventional semiconductors, however, hot carriers typically lose energy quickly, thermalizing to the conduction band minima (CBM) on sub‐picosecond timescale. The initial hot carrier thermalization is dominated by carrier‐carrier and carrier‐phonon scattering in 100 fs timescale, followed by energy loss through interactions with longitudinal optical (LO) phonons over several picoseconds and with longitudinal acoustic (LA) phonons on the order of ∼100 ps [43].

The initially generated hot excitons can rapidly dissociate into free carriers, which subsequently interact with optical phonons [45]. Both excitons and free carriers coexist in MAPbBr_3_ at room temperature, with the free‐carrier population increasing as thermal energy promotes exciton dissociation [46, 47]. One of the primary mechanisms responsible for the prolonged lifetime of hot carriers is the formation of polarons (Figure 4b), arising from the coupling of excited carriers with lattice vibrational modes [48]. Although both excitons and free carriers coexist in MAPbBr_3_, the measured PL broadening reflects the spatial redistribution of the photoexcited electron–hole population through radiative recombination and is therefore representative of ambipolar carrier transport. Since large‐polaron formation requires free carriers rather than bound excitons, the observed super‐diffusive transport is expected to be governed primarily by the free‐carrier population, while the measured PL expansion reflects the collective transport of the photoexcited carriers. In metal halide perovskites, large polarons play a particularly important role: carriers interact with the vibrational modes of the inorganic sub‐lattice over several unit cells through long‐range Fröhlich interaction [49]. The formation of such large polarons at room temperature suggests that they are unconventional in nature, influenced by anharmonic phonons due to a soft crystal structure, and dipolar cations showing Debye relaxation [50, 51]. Unlike small polarons, which leads to localized carriers that migrate via thermally activated hopping, large polarons support band‐like, coherent carrier diffusion [52, 53]. Polaron formation is known to occur shortly after the initial thermalization of hot carriers [54]. These large polarons participate actively in the subsequent cooling of the hot carriers by absorbing the excess energy [55]. Their increased effective mass also screens carriers from scattering with defects, impurities, and phonons, thereby suppressing the nonradiative recombination pathways and slowing hot carrier cooling [53, 56]. In addition to enabling long‐range carrier migration, polarons have been proposed to mitigate ion‐migration‐induced degradation by altering the energy landscape.

Further, the interaction between the hot carriers and accessible vibrational states can give rise to a hot‐carrier bottleneck, in which a non‐equilibrium population of phonons builds up, storing excess energy [57]. Under these conditions, the carrier‐phonon scattering rate is reduced, and phonon cooling to acoustic modes becomes inefficient. Strong coupling between excited carriers and phononic states may also lead to resonant interactions with specific vibrational modes, particularly the longitudinal optical (LO) phonons (Figure 4c). Identifying pathways to stabilize hot carriers remains a major objective in the perovskite research community and has been actively pursued through both theoretical and experimental investigations [58, 59, 60]. Significant efforts have been devoted to identifying the mechanisms that govern hot‐carrier cooling and stabilization, as well as to extracting hot carriers prior to thermalization losses, with the aim of improving the efficiency of next‐generation photovoltaic and optoelectronic devices [61, 62].

Of the mechanisms proposed to govern the hot‐carrier stabilization, each dominates within a distinct carrier density regime. Large polaron formation is favored at a carrier densities below ∼10^18^ cm^−3^, while the hot‐phonon bottleneck is generally considered a dominant mechanism at densities exceeding ∼10^18^ cm^−3^. Notably, large polaron formation and hot carrier stabilization have been observed in MAPbBr_3_ even at relatively low carrier densities (<10^17^ cm^−3^) [63]. The excitation power used here (∼1 fJ/pulse, corresponds to a carrier density of ∼2 × 10^16^ cm^−3^) corresponds to a carrier density below 10^17^ cm^−3^, well within the regime where polaron formation is expected to dominate. The observed carrier super‐diffusion shows only a weak dependence on excitation power (Figure S6), with the degree of non‐linearity occasionally decreasing with decreasing laser power, likely due to crystal‐to‐crystal heterogeneity. Furthermore, the relatively low carrier densities employed here make a significant contribution from the hot‐phonon bottleneck mechanism unlikely, as this process is generally reported to become prominent at substantially higher carrier densities [41, 43]. We therefore conclude that the primary mechanism responsible for slow hot carrier cooling in these MAPbBr_3_ MDs is the formation of large polarons, potentially complemented by additional processes involving interactions between excitonic states and optical phonic modes. It is important to distinguish the timescale of large‐polaron formation from the timescale over which its influence on carrier transport persists. While large polarons form on sub‐picosecond to picosecond timescales, the observed nanosecond‐scale super‐diffusion likely reflects a dynamic carrier–phonon environment in which ongoing interactions between photoexcited carriers and lattice vibrations continue to influence carrier scattering and transport.

The strong coupling and equilibrium between hot carriers and LO‐phonons are enabled by the low LO‐phonon frequency and the low electronic density of states in MAPbBr_3_ [50, 64]. The closely spaced LO states (∼15 meV) facilitate resonance with accessible excited electronic states. Hot carrier cooling in this system is therefore likely governed by multiple inter‐connected mechanisms that involve both LO phonons and large polaron formation. Because population of the vibrational modes is temperature sensitive, the degree of super‐diffusivity should depend on the rate at which energy is exchanged within the strongly coupled carrier – LO phonon system. To probe the effect of temperature, we measured the carrier diffusion behavior of an MD at room and slightly elevated temperatures. At 23°C the MD exhibited typical super‐diffusive carrier transport, with *D*_ini_ = 0.42 cm^2^/s, *D*_equ_ = 0.74 cm^2^/s, and α = 1.39 (Figure 4d–f). Remarkably, increasing the temperature by only 4°C led to a much more rapid broadening of the PL profile. This enhancement is evident in both the PL cross‐section evolution (Figure 4h) and the MSD plot (Figure 4i), where the diffusion accelerates from the initial value *D*_ini_ = 0.37 cm^2^/s to an exceptionally high *D*_equ_ = 1.96 cm^2^/s. The non‐linearity coefficient also increases dramatically to α = 2.32, an unexpectedly large change for such a modest rise in temperatures.

The strong enhancement of super‐diffusivity induced by a modest 4°C increase in temperature is surprising and was consistently observed across multiple measurements (Figure S7). Furthermore, the diffusivity is measured same MD was measured again after returning the temperature from 27°C to 23°C. The MSD evolution and extracted transport parameters recovered to values comparable to those obtained before heating (Figure S8), indicating that the enhanced super‐diffusive transport is reversible within the experimental uncertainty. No irreversible changes in the PL image of the crystal or in the carrier transport behavior were observed following the temperature cycle. Although the LO phonon energy itself is weakly affected by temperature, the population of higher vibrational states can increase to a certain extent. Moreover, the corresponding increase in the free‐carrier population is expected to be relatively small under these conditions. While these observations highlight the important role of carrier–phonon interactions in the super‐diffusive transport process, the precise origin of the pronounced temperature dependence remains unclear and warrants further investigation. These results suggest that subtle temperature changes in phonon occupation may fine‐tune resonance conditions between the LO modes and the hot electron states, thereby producing a sharp rise in super‐diffusivity. Elevated temperatures may thus enhance the carrier‐phonon coupling pathways that facilitate accelerated hot carrier transport.

## 4 Conclusion

In summary, we discovered super‐diffusive carrier transport in MAPbBr_3_ micro‐disks grown under anti‐solvent ambiance. The phenomenon is characterized by rapid outward migration of hot carriers and pronounced non‐linear spatial PL broadening in time. Deep‐band excitation generates long‐lived hot carriers which, through strong coupling with LO phonons, undergo super‐diffusive propagation on nanosecond timescales. This behavior is evidenced by diffusivities increasing over time, high non‐linearity coefficients exceeding those of free diffusion by more than 50%, and hot‐carrier migration lengths extending hundreds of nanometers within a few nanoseconds. Comparisons with longer wavelength excitation and temperature‐dependent measurements confirm that the extent of super‐diffusion is governed by the energetics of hot‐carrier generation and the phonon occupation in the lattice. The results support a mechanism primarily driven by large‐polaron formation, with additional possible contributions from resonant LO phonon interactions that modulate carrier energy loss pathways. The discovery of nanosecond‐scale super‐diffusion in perovskite microstructures expands the current understanding of carrier dynamics in these materials and highlights the role of hot‐carrier stabilization beyond the picosecond regime traditionally associated with metal halide perovskites. These insights have important implications for hot‐carrier photovoltaics, where minimized thermalization‐induced energy loss relies on controlled management of hot‐carrier transport and cooling pathways.

## Author Contributions

**Nithin Pathoor**: conceptualization, investigation, writing – original draft, methodology, writing – review and editing. **Martin Vacha**: writing – review and editing, project administration, funding acquisition, supervision. **Shun Omagari**: investigation, formal analysis.

## Funding

The research was financially supported by the JSPS KAKENHI grant number 24K01449 and by the JSPS KAKENHI grant number 23H04875 in Grant‐in‐Aid for Transformative Research Areas “Materials Science of Meso‐Hierarchy”.

## Conflicts of Interest

The authors declare no conflicts of interest.

## Supporting information




**Supporting File**: advs76474‐sup‐0001‐SuppMat.pdf.

## Data Availability

The data that support the findings of this study are available from the corresponding authors upon request.
