# Supplementary material for: Efficient Hot Carrier Management in Lead Halide Perovskite Micro‐Disks Revealed by Super‐Diffusive Migration
Source: Adv Sci (Weinh). 2026 Jul 13:e76474. Online ahead of print. doi: 10.1002/advs.76474 (PMC13359405; doi:10.1002/advs.76474)
Supplement: Supplementary file 1 — Supporting File: advs76474‐sup‐0001‐SuppMat.pdf. [file ADVS-9999-e76474-s001.pdf]

Supporting Information

Efficient hot carrier management in lead halide perovskite micro-disks revealed by super-diffusive migration

Nithin Pathoor, Shun Omagari, Martin Vacha**

Materials and methods

Sample preparation:

Methyl ammonium bromide and lead bromide were purchased from Sigma-Aldrich. DMF and toluene are purchased from Sigma-Aldrich and Kanto Chemical co. inc. respectively. All chemicals were used as such. A 0.05 M precursor solution of $MAPbBr_3$ was prepared by dissolving 11.2 mg of MABr (MW = 111.97) and 36.7 mg of $PbBr_2$ (MW = 367.01) in 2 ml of DMF. The solution was stirred till the coverslips are UV-ozone cleaned for 30 mins. A small Petri dish was placed inside a beaker upside down, and ~6 ml of toluene is added to the big beaker without submerging the Petri dish. The ozone cleaned coverslip was placed on the Petri dish and 50 μl of precursor solution was added on top. The beaker is covered with perforated aluminium foil and placed inside drawer for ~24 hours. The distance between the substrate and the toluene level was ~ 1 cm.

Characterization:

Field Electron Scanning electron microscopy (FE-SEM) was performed using a JSM-7500F instrument (JEOL Ltd.). The absorption spectra of the thin film were measured using a UV-Vis spectrophotometer (Jasco, V-760) and the PL emission spectra were measured using Jasco FP-6200 Spectrofluorometer.

Fluorescence microscopy and measurement of excited carrier diffusivity:

The fluorescence imaging, spectroscopy and diffusion measurements were performed with a widefield epi-fluorescence microscope (Olympus, IX73) custom-modified for confocal excitation and spatially resolved lifetime measurements.

A 375 nm pulsed laser (PicoQuant, LDH-D-C375 laser controlled via PDL 800-D) with 10 MHz repetition rate was focussed into the back-focal plane of an oil immersion objective (Olympus, 1.3 NA, 100x) to achieve widefiled illumination. A dichroic beam splitter (Semrock, FF376-Di01-25x36) and an emission filter (Semrock, FF01-380/LP-25) is used the filter cube of the microscope. The sample was confocally illuminated by collimating an expanded laser beam into the objective lens, with an excitation power of ~0.9 fJ/pulse. The emission is collected with the same objective, and a pair of convex lenses (f =7 cm) are used as relay lenses in the emission path of the microscope. The PL images were collected with an EMCCD camera (Andor iXon3), with an attached monochromator (CLP-50LD, Bunkou Keiki) for spatially resolved spectroscopic measurement.

For spatially resolved lifetime measurement, a flipper mirror is used between the relay lenses to reflect the emission into the avalanche photodiode (Micro Photon Devices SPD-050-CTC) through a long focal length convex lens (f = 30 cm). This resulted in a magnification of 431x in the APD detection plane. The APD is placed on a 1D piezo-stage, and PL lifetime was measured (with 16 ps time binning) at locations across the excitation spot by scanning the APD with 50 μm x 50 μm (equivalent to ~116 nm x 116 nm) active area at 30 μm intervals (equivalent to ~70 nm steps in sample plane). We used HydraHarp 400 multi-channel picosecond event timer from PicoQuant for lifetime measurements.

Elevated temperature experiment was performed using an objective heater assembly from Bioptechs, USA.

Estimation of carrier density

The excitation pulse energy used in the experiments was approximately 1 fJ/pulse. A confocal excitation spot with a full width at half-maximum (FWHM) of ~500 nm and a crystal thickness of ~500 nm yields an excitation volume of approximately 0.1 μm^3 (1×10^{-13} cm^3). The number of photons per pulse at 375 nm is estimated to be ~2×10^3. Assuming nearly complete absorption of the incident photons due to the crystal thickness exceeding 500 nm, the generated carrier density is estimated to be ~2×10^{16} cm^{-3}. To achieve a comparable carrier density under 485 nm excitation, an excitation pulse energy of 0.8 fJ/pulse was used.

Calculation of Diffusion coefficient and diffusion length:

A 2D map of the intensity cross-section as a function of delay time is generated from spatially resolved lifetime data. The PL intensity cross-section, binned to 80ps is fitted with a Gaussian

function and variance is calculated. The mean square displacement is calculated, and a fitting model is applied according to the equation,

$MSD = \sigma(t)^2 - \sigma(0)^2 = 2Dt^{\alpha}$, where D is the diffusion coefficeient and α is the non-linearity coefficient. For free-diffusion, α=1, whereas α>1 indicates super-diffusive transport.

The initial non-linear regime of the MSD evolution was fitted using the generalized diffusion model described above to extract the initial diffusion coefficient (D_{ini}) and diffusion exponent (α). Because the duration and extent of the super-diffusive behavior vary among individual MDs, the transition between the non-linear and linear regimes is not uniquely defined. Therefore, the fitting windows were selected individually for each MSD curve. After the initial super-diffusive regime, the approximately linear portion of the MSD curve was identified and fitted using the linear diffusion relation, MSD=2Dt, to determine the equilibrium diffusion coefficient (D_{equ}).

The diffusion length is calculated with the formula, $L_D = \sqrt{\tau_{av} D_{equ}}$.

The bulk PL lifetime is used to accurately calculate diffusion length $L_D = \sqrt{\tau_{bulk} D_{equ}}$.

The transport length is defined as,

$l_t = \sqrt{\sigma(t)^2 - \sigma(0)^2}$

The transport length (l_{t}) exhibits an approximately linear increase within the super-diffusive regime, followed by a transition to a slower diffusion regime. The l_{t} trajectories were fitted with two linear functions, and their intersection was used to determine the characteristic super-diffusive timescale (t_{sd}) and transport distance (d_{sd}).

All analysis were performed with Matlab and OriginLab.

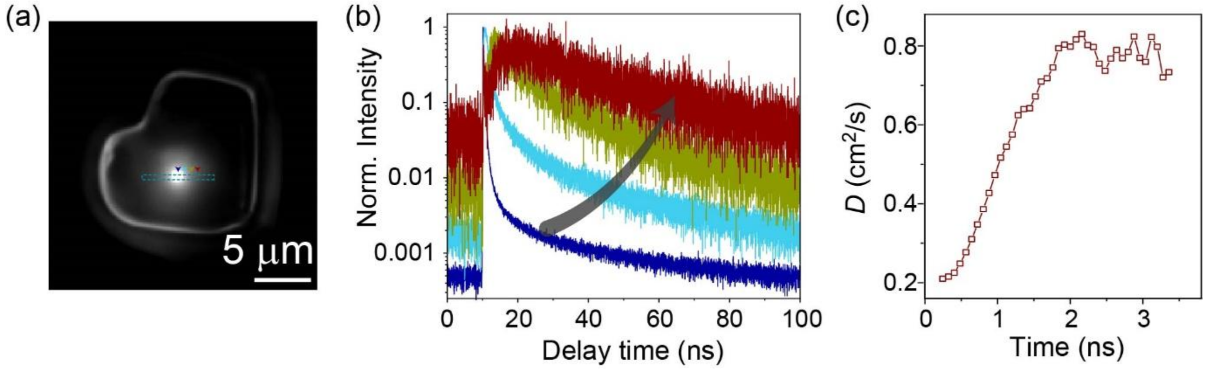

Figure S1. (a) PL image of a MD with confocal excitation (b) Fluorescence decay curves at the excitation location and position away from it at an interval of ~500 nm. The decay of fluorescence intensity slows down as we move away from the excitation location. At 1 micron and 1.5 micron distance, a raise of emission intensity can be observed after photoexcitation. (c) Temporal evolution of effective diffusion coefficient calculated through tangent of MSD plot.

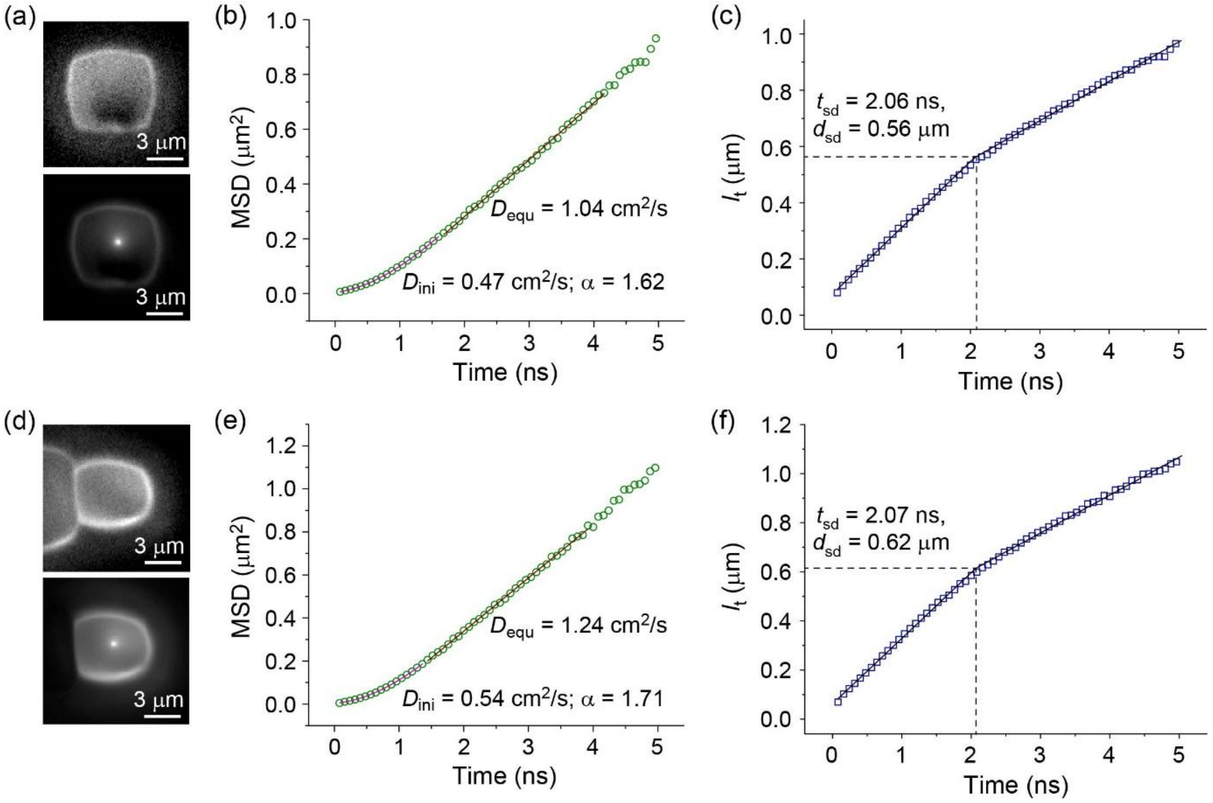

Figure S2. Super-diffusive carrier diffusion of two representative MDs. (a, d) Widefield (top) and confocal (bottom) PL images. (b, d) MSD evolution and transportation length curves of the two MDs.

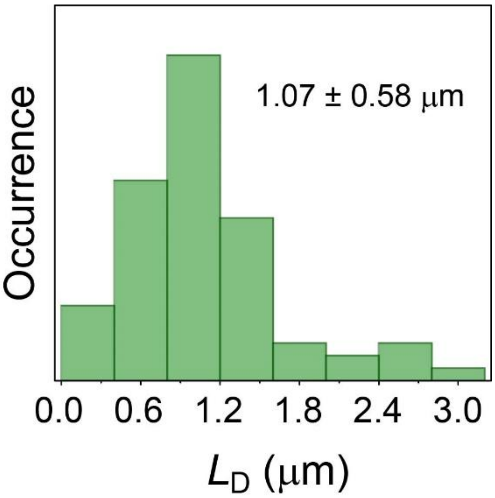

Figure S3. Distribution of carrier diffusion length estimated with equilibrium diffusion coefficient and excitation location lifetime. As the lifetime is shorter at the excitation location, the calculated diffusion coefficent is under-estimated.

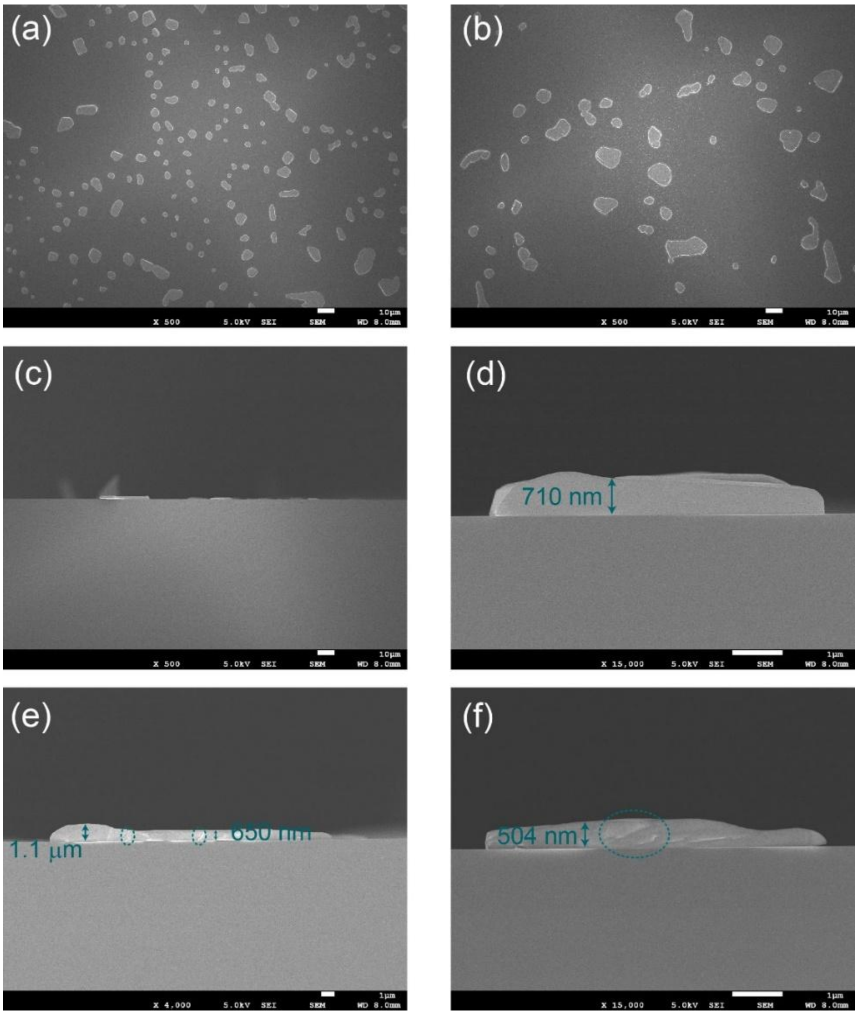

Figure S4. (a-b) Representative SEM images of $MAPbBr_3$ thin film showing the distribution of size and shapes of mesa-shaped MDs. (c-f) The cross-sectional SEM imgaes depicting the thickness of MDs and heterogeneity in the crystal quality among different crystals.

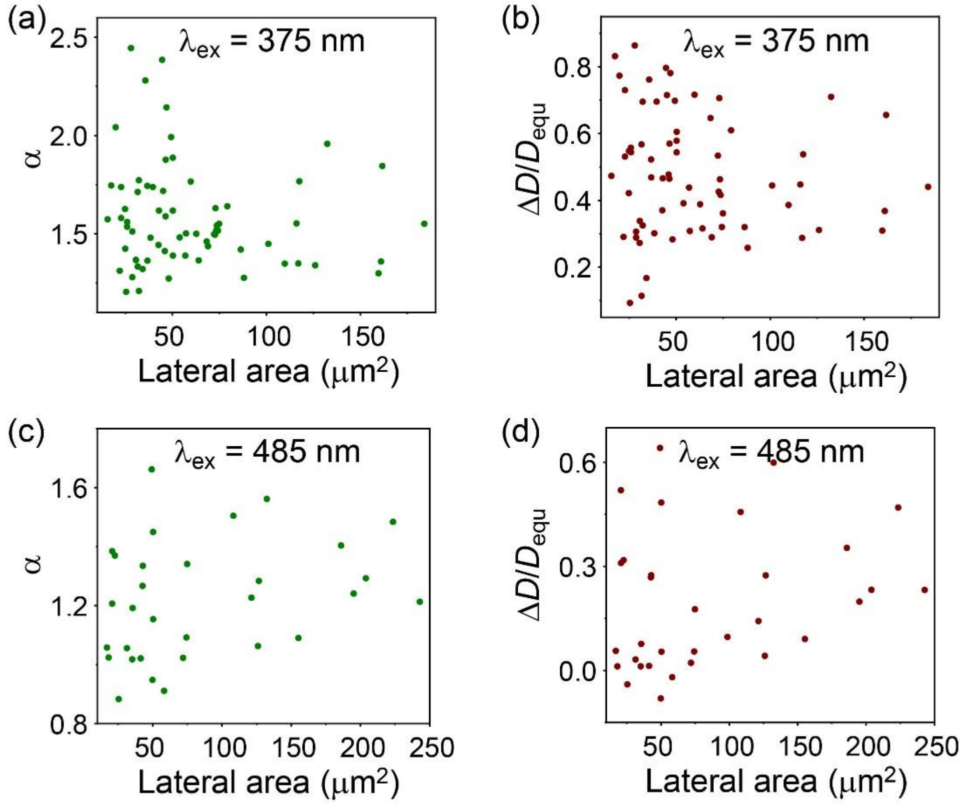

Figure S5. The lateral dimensiton dependence of defree of super-diffusivity under 375 nm and 485 nm excitation.

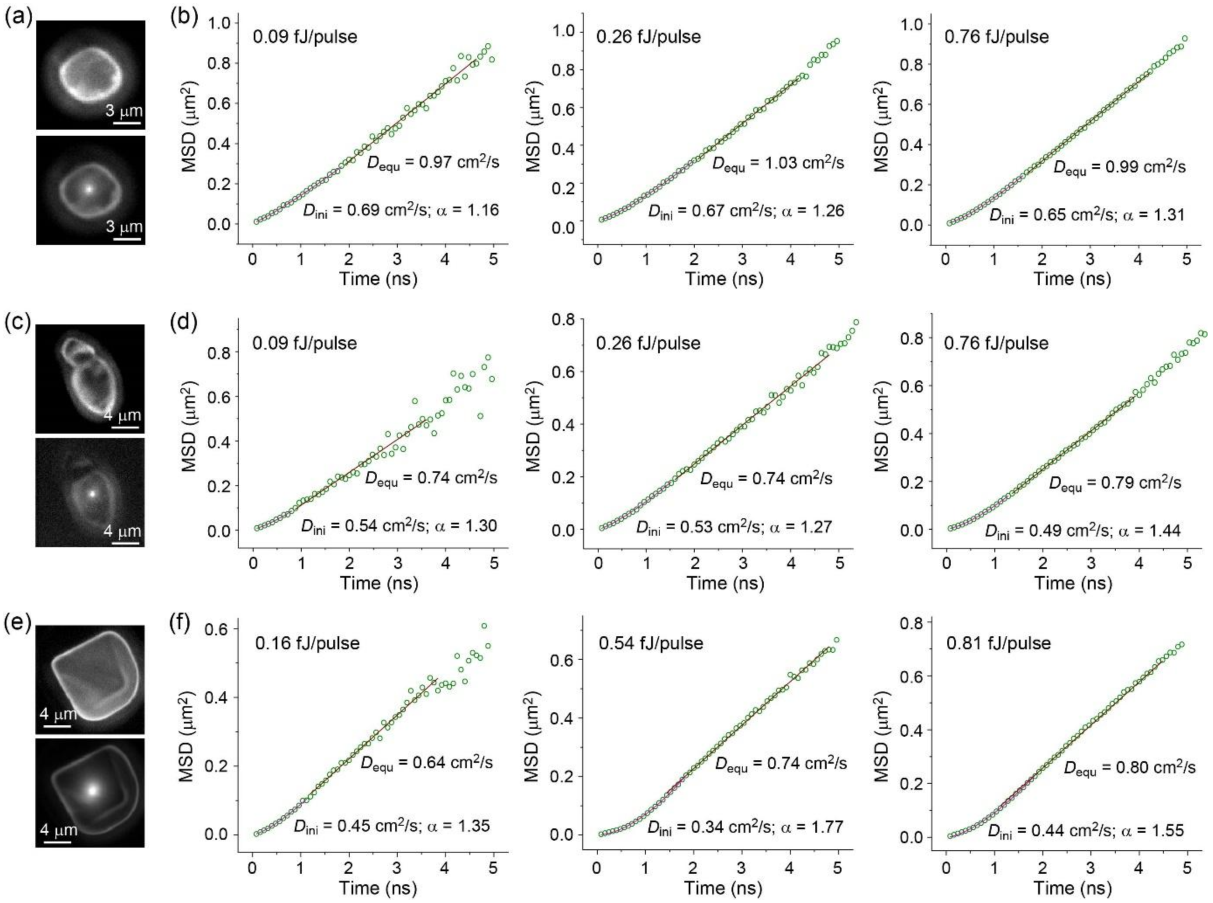

Figure S6. Excitation power dependence of carrier diffusion. (a, c, e) PL images and (b, d, f) MSD evolution curves for three MDs at different laser powers.

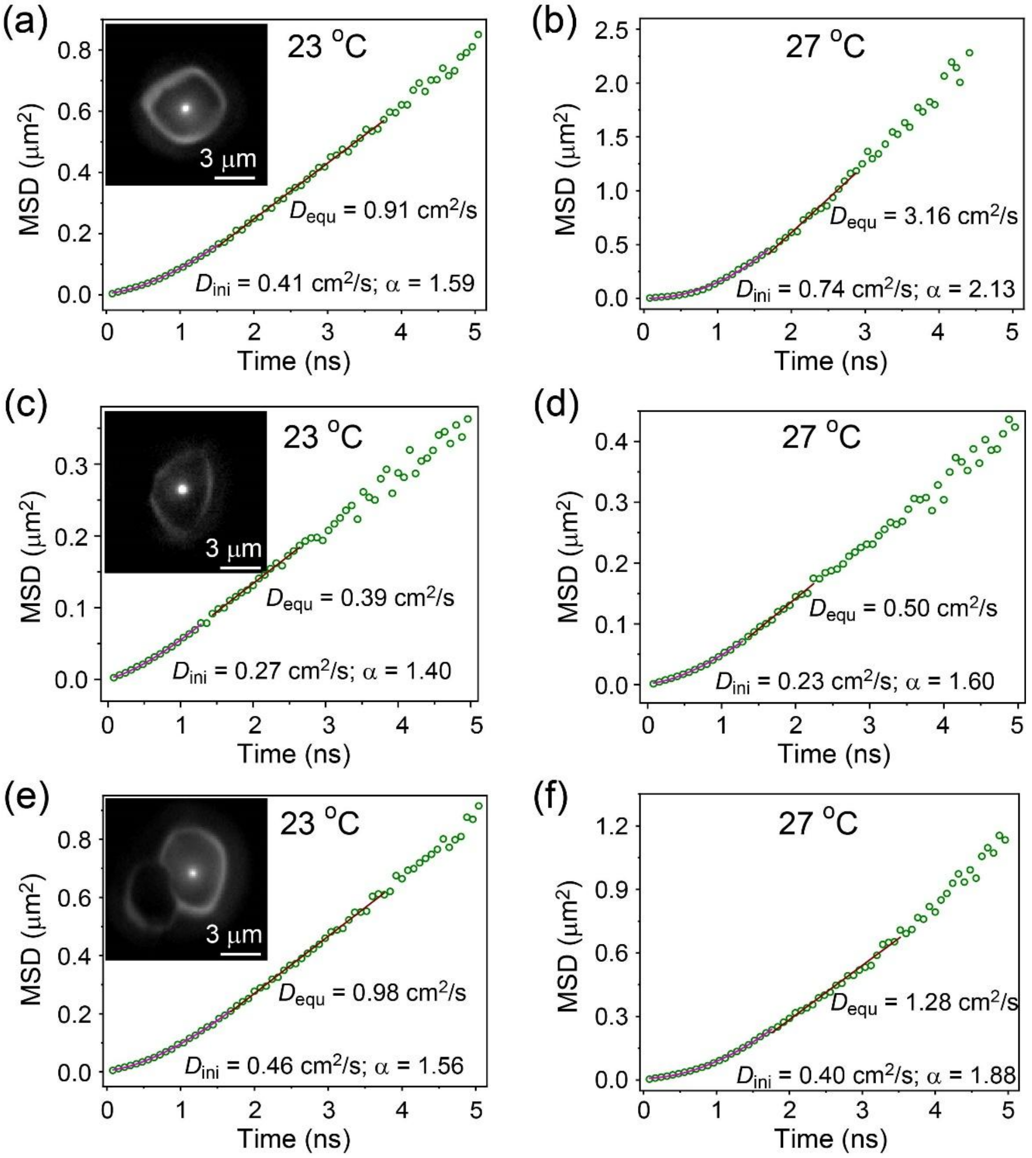

Figure S7. The time evolution of MSD at room temperature, 23 °C (a, c, e) and 27 °C (b, d, f) of three MDs.

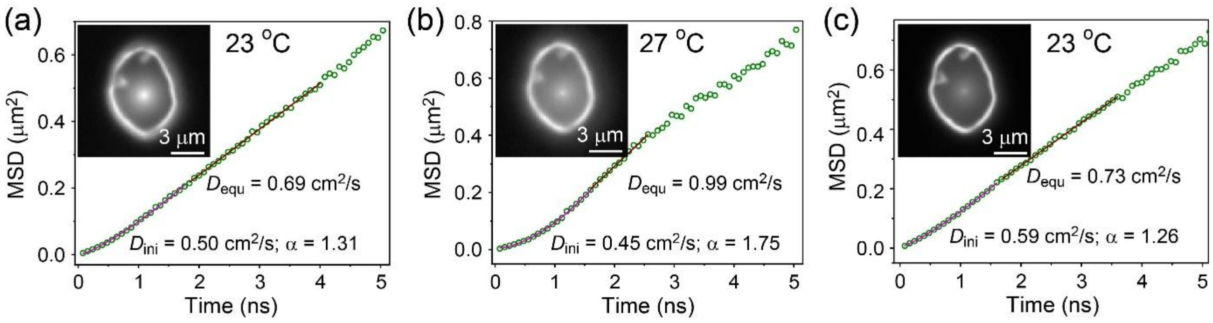

Figure S8. MSD plots of a representative MD measured at (a) room temperature (23 °C), (b) elevated temperature (27 °C), and (c) after returning to room temperature (23 °C).
